# Irradiation of the kidneys causes pathologic remodeling in the nontargeted heart: A role for the immune system

**DOI:** 10.1096/fba.2020-00071

**Published:** 2020-10-23

**Authors:** Marek Lenarczyk, Evagelia C. Laiakis, David L. Mattson, Bryon D. Johnson, Amy Kronenberg, Paula E. North, Richard Komorowski, Marylou Mäder, John E. Baker

**Affiliations:** ^1^ Medical College of Wisconsin Milwaukee Wisconsin USA; ^2^ Georgetown University Washington D.C USA; ^3^ Medical College of Georgia Augusta Georgia USA; ^4^ Lawrence Berkeley National Laboratory Berkeley California USA

**Keywords:** cytokines, Fibrosis, heart diseases, hypertension, rats, T lymphocytes, X‐rays

## Abstract

Cardiac disease is a frequent and significant adverse event associated with radiotherapy for cancer. Identifying the underlying mechanism responsible for radiation injury to the heart will allow interventions to be developed. In the present study, we tested if local kidney irradiation results in remodeling of the shielded, nontargeted heart. One kidney, two kidneys, or the total body of male WAG and Dahl SS rats were irradiated with 10 Gy of X‐rays. Local kidney irradiation resulted in systemic hypertension, increased BUN, infiltration of T lymphocytes, natural killer cells, and macrophages into the renal cortex and medulla, and renal fibrosis. Local irradiation of kidneys in WAG rats resulted in remodeling in the nontargeted heart after 120 days, manifested by perivascular fibrosis and increased interventricular septal thickness, but was not seen in Dahl SS rats due to a high baseline level of fibrosis in the sham‐irradiated animals. Genetic depletion of T cells mitigated the nephropathy after local kidney irradiation, indicating a role for the immune system in mediating this outcome. Local kidney irradiation resulted in a cascade of pro‐inflammatory cytokines and low‐molecular weight metabolites into the circulation associated with transmission of signals resulting in pathologic remodeling in the nontargeted heart. A new model is proposed whereby radiation‐induced cardiac remodeling in susceptible animals is indirect, with lower hemi body organs such as the kidney exporting factors into the circulation that cause remodeling outside of the irradiated field in the shielded, nontargeted heart. This nontargeted effect appears to be mediated, in part, by the immune system.

**Abbreviations**
AOC: area under the curve
BUN: blood urea nitrogen
Dahl SS: SS/JrHsdMcwi
FDR: false discovery rate
Gy: Gray
IHC: immunohistochemistry
MCW: Medical College of Wisconsin
MS: mass spectrometer
RAAS: renin–angiotensin–aldosterone system
ROC: receiver operating characteristic
TBI: total body irradiation
WAG: WAG/RijCmcr

## 1 BACKGROUND

Radiation is a cornerstone of successful cancer treatment, with over one‐half of all cancer patients receiving radiotherapy.^1^ Survivorship in cancer patients treated with therapeutic radiation is increasing. In 2016, there were an estimated 3.05 million cancer survivors who had been treated with radiation therapy. The number of radiation‐treated cancer survivors is projected to reach 3.38 million in 2020, and 4.17 million by 2030.^2^ Survivors of childhood and adolescent cancers who received direct cardiac irradiation with 15 Gy or more as part of their treatment are at increased risk for heart disease ^3^, ^4^. Irradiation of the lower hemi body below the diaphragm, but not upper hemi body irradiation, with 10 Gy of X‐rays, increases risk factors for cardiac disease and results in cardiac fibrosis much like that which has been observed in rats given TBI ^5^, suggesting radiation‐induced injury to the heart can be indirect. This finding suggests a new research paradigm where radiation‐induced cardiac pathology can be indirect, with abdominal organs generating factors that cause cardiac remodeling.

The kidney is known to be a highly radiosensitive organ susceptible to the development of nephropathy, proteinuria, and hypertension after irradiation.^6^ Historically, pelvic or abdominal radiotherapy resulted in exposure of kidneys in the irradiation field to a dose that could reach 10 Gy,^7^ a dose that is known to cause renal injury.^8^, ^9^ Renal dysfunction has been proposed as part of the mechanism causing increased cardiac disease in cancer survivors treated with TBI^10^ and in survivors of atomic bombs.^11^ Dysfunction in the kidney inducing cardiac dysfunction was described as early as 1836 by Richard Bright.^12^ Shielding of the rat kidneys during irradiation prevents the increase in risk factors for cardiac disease and kidney injury in this experimental animal model, indicating the importance of the kidney in mechanisms that underlie radiation‐induced cardiac disease.^5^ These findings suggest injury to the heart from irradiation can be indirect, supporting the notion that injury to an abdominal organ such as the kidney plays a role in the occurrence of cardiac disease after irradiation below the diaphragm. This hypothesis can be directly tested by locally irradiating the kidney and determining whether there is disease in the nontargeted heart. Nontargeted effects manifest as cellular responses in cells in which ionizing radiation has not been deposited.

There is no receptor specifically engaged by ionizing radiation in mammalian systems, nor are there unique mechanisms identified that directly detect ionizing radiation exposure. The innate immune system acts as the sentinel to sense tissue damage from radiation and alert the body.^13^, ^14^ Activation of the immune system can lead to the production of pro‐inflammatory cytokines with corresponding increases in the circulation.^15^

The existence of a mechanism linking local radiation injury of kidney to heart disease requires the transmission of a signal between the two organs. Injury to the nontargeted heart would occur at a distance from the irradiated kidney and would be independent of any direct exposure of the heart to radiation.^16^ Evidence of such a remote effect after radiotherapy of abdominal organs, such as the kidney, would be manifested by remodeling of the distant heart. Understanding how the immune system responds to irradiation of normal healthy tissue is key to establishing the underlying mechanism of injury. To our knowledge, an active role for the immune system in the response to local irradiation of the kidney that affects distant organs such as the heart has not yet been demonstrated.

We tested the hypothesis that local irradiation of the kidney remodels the nonirradiated (i.e., nontargeted) heart. The major objectives were to determine: (a) the extent of renal injury and cardiac remodeling after irradiation of one kidney, two kidneys, or the whole body, (b) the extent of immune cell activation in kidneys following local kidney irradiation, (c) the identity of the signaling molecules originating from the kidney after irradiation, and (d) the role of the adaptive immune system in the genesis of radiation nephropathy. Our study demonstrates that local kidney irradiation can result in T cell activation that is associated with pathologic remodeling in the nontargeted heart in a susceptible rat strain.

## 2 METHODS

### 2.1 Compliance with design and statistical analysis requirements

Group sizes were equal. Power analysis was used to determine that eight rats per group were needed to detect the anticipated effect size based on outcomes in previous studies at a significance level of 0.05 with a power level of 0.80. Animals were randomized to each group. The identity of animals in each experimental group was known to the investigator responsible for initiating and continuing with an intervention, for example radiation exposure. Identity of the animal under study was not known to the investigator performing the experimental measurement or the analysis. These investigators were not the same person.

### 2.2 Experimental animals

Male WAG, Dahl SS with a null deletion in *CD247* (CD3 ζ) (Dahl SS CD247^−/−^) rats, and Dahl SS wild‐type littermates (Dahl SS CD247^+/+^) rats were used for these studies which commenced at 6‐8 weeks of age. Animals were pair housed and maintained on rat chow (Teklad 8904) and water *ad libitum* in the Biomedical Resource Center of the MCW, Milwaukee, Wisconsin, USA. The rats were maintained on a 12‐hour light cycle, 12‐hour dark cycle at a temperature of 20 ± 1°C, and relative humidity 50%–80%. The drinking water supplied to the rats was further purified by reverse osmosis and then chlorinated. The water the rats drank was not acidified. Animal studies were conducted in compliance with the US National Research Council's Guide for the Care and Use of Laboratory Animals, the US Public Health Service's Policy on Humane Care and Use of Laboratory Animals, and Guide for the Care and Use of Laboratory Animals, in accordance with NIH guidelines. The Animal Care and Use Committee at MCW approved all experiments involving live animals. The start of the study was defined as the time rats were irradiated or sham treated.

### 2.3 Irradiator and dosimetry

Irradiations were conducted with orthovoltage X‐rays from an X‐RAD 320 X‐ray unit (Precision X‐Ray Inc, Branford, CT) and a Pantak HF320 orthovoltage system (Therapax, Danbury, CT) at the MCW Radiation Core facility. The radiation dosimetry has been described previously,^17^

### 2.4 Posterior–anterior irradiation

To test whether local irradiation of one kidney, both the kidneys or the total body with a single dose of 10 Gy in WAG rats resulted in nephropathy and remodeling in the heart, a posterior–anterior field was used initially. The adrenals, parts of the liver, and the intestines were also within the trajectory of the collimated radiation beam during irradiation of one kidney or both the kidneys. The rest of the body was shielded using a 1/3 inch thickness lead plate. Conscious WAG rats (n = 8/group) were immobilized in acrylic plastic jigs during irradiation. Animals were oriented in the beam line with their sides perpendicular to the beam to ensure uniform irradiation throughout the rat. The dose rate was 1.27 Gy/min for irradiation of one kidney, 1.32 Gy/min for irradiation of both the kidneys, and 1.73 Gy/min for irradiation of the total body. The X‐ray machine was operated at 300 kVp with a half‐value layer of 1.4 mm copper. The need to use bone marrow transplantation in animals that received TBI, which was used in a previous study,^17^ was avoided by shielding the bone marrow in one leg using a 1/3 inch thickness lead plate. This had no effect on the progression of cardiac injury. Rats were irradiated and maintained in the animal care barrier facility at MCW throughout the study. Sham‐irradiated rats (n = 8/group) served as controls.

### 2.5 Lateral irradiation

To test whether local irradiation of both the kidneys resulted in remodeling of the nontargeted heart in all subsequent studies in WAG and Dahl SS rats, a lateral field was used to irradiate the kidneys avoiding irradiation of the liver and intestines. Conscious rats were immobilized in acrylic plastic jigs during irradiation with parallel‐opposed lateral fields that included both the kidneys.^17^ The adrenals were also exposed to radiation. The rest of the body was shielded using a 1/3 inch thickness lead plate. Animals were oriented in the beam line with their sides perpendicular to the beam to ensure uniform irradiation throughout the rat. The anatomic location of the kidneys was confirmed using a PaxScan 2520 V Amorphous Silicon Digital X‐Ray Imager (Varian) attached to X‐RAD320 X‐ray unit to capture images of the exposed field. Rats received local kidney irradiation using a collimated beam with a total of 10 Gy resulting from two fractions of 5 Gy with the rat rotated once 180 degrees halfway through the irradiation procedure to ensure appropriate dose uniformity because of the anatomical location of the kidneys. Sham‐irradiated rats were immobilized in acrylic plastic jigs for the same amount of time required for an average irradiation served as controls. The dose rate for the local kidney irradiation studies was 1.29 Gy/min for WAG rats and 1.26 Gy/min for Dahl SS rats. The X‐ray machine was operated at 300 kVp with a half‐value layer of 1.4 mm copper.

#### 2.5.1 Kidney injury and cytokine measurement

Blood was obtained from the jugular vein of WAG and Dahl SS rats at 20, 40, 60, 80, 100, and 120 days after TBI alone, after local kidney irradiation, and from sham‐irradiated control rats. Blood collection continued at 150, 180, and 210 days in Dahl SS rats. Serum was analyzed for BUN, total protein and albumin levels (Wisconsin Diagnostic Laboratories). Serum samples were then analyzed to determine the concentration of 27 cytokines (Eve Technologies).

#### 2.5.2 Systemic blood pressure

Systemic blood pressure in trained and conscious rats was measured using a tail pressure cuff as described elsewhere.^17^

#### 2.5.3 Metabolomic studies

Serum samples were obtained from male WAG rats for untargeted metabolomic analysis at 20, 30, 40, and 50 days after irradiation of both the kidneys and compared to sham‐irradiated, age‐matched samples. Serum samples were diluted 1:40 in 66%:34% acetonitrile:water, vortexed, and incubated on ice for 10 minutes, followed by a 10 minutes centrifugation step at 4°C at 13 000 × *g* to precipitate the protein. The supernatants were transferred to a clean mass spectrometry vial and 2 μL was injected into a BEH C18 column (130 Å, 1.7 μm, 2.1 × 50 mm, 60°C) on an Ultra Performance Liquid Chromatography system (UPLC^®^) coupled to a Xevo^®^ G2 time‐of‐flight MS (Waters, Milford MA). The chromatographic conditions have been described previously.^18^, ^19^ Quality controls from pooled samples were injected every 10 samples. Chromatographic alignment, peak picking, and deconvolution of the data were conducted with the software Progenesis QI (NonLinear Dynamics). Normalization of the data was performed with the option “Normalize to all compounds” to create a normal distribution of the data as previously described.^19^ Analysis was conducted in both ESI+ and ESI− in MS^E^ mode. Data analysis and biomarker discovery were conducted with the software MetaboLyzer^20^ and MetaboAnalyst.^21^ All conditions in the analysis were identical to previously described procedures,^22^ except that Mann–Whitney *U* test and Barnard's statistical tests were used for biomarker identification, with a FDR of 0.2. A principal component analysis scores plot of the ESI‐ data was generated from the normalized data with the software SIMCA‐P^+^ v. 13.0.3 (Umetrics, Umea Sweden) of the first two components, [t1] and [t2].

Positive identification of potential biomarkers was conducted with tandem mass spectrometry (MS/MS), where fragmentation patterns of both experimental samples and pure chemicals were matched and cross‐referenced to experimental spectra in the METLIN database. Multivariate ROC curves were constructed from 12 validated metabolites through MetaboAnalyst, with Random Forests as the classification method and feature ranking method. An AUC value of 0.8‐0.9 was considered good, while an AUC of 0.9‐1 was considered excellent. Significant features were ranked by their mean importance measure. Graphical representation of individual metabolites was conducted with the software Prism 6 (GraphPad) that allowed also for statistical analysis. A Student's *t* test was conducted for each time point with a FDR correction of Q = 10%. All data were represented as mean ±standard error of the mean, with a *P*‐value of <.05 considered statistically significant.

#### 2.5.4 Histology

To evaluate tissue damage following irradiation, the entire heart and both the kidneys were isolated from fully anesthetized irradiated and sham‐irradiated (control) rats and fixed in 10% formalin (v/v) using our standard procedures as described elsewhere.^17^ We consistently obtained traverse sections of heart tissue from the middle of both ventricles. For the kidney, longitudinal sections were obtained for cortex and medulla. Prior to embedding, the heart was oriented between the base and the apex, and the kidneys oriented along the mid‐dorsal plane. Fixed tissue samples were embedded in paraffin with kidney samples in coronal orientation and heart samples in the transverse plane. Sections 4‐µm thick were cut from each block and stained with Masson's trichrome blue according to standard methods described elsewhere.^17^ Ten sections from each heart and kidney were used for morphometric analysis as described elsewhere.^17^ Cardiac fibrosis was defined as an increase in perivascular cardiac collagen content above values for sham‐irradiated animals.^5^

#### 2.5.5 Immunohistochemistry

Paraffin‐embedded sections were stained immunohistochemically using a DAKO Autostainer Plus automated staining platform. Antibodies included: T cells with DAKO Rabbit Polyclonal CD3^+^ (A0452, 1:100), natural killer cells with Cell Marque mouse monoclonal CD56^+^ (156R‐94, 1:200), and macrophages with EMB Millipore mouse monoclonal (CD68^+^ (MAB1435, 1:100). Pretreatment was performed with citrate buffer for CD56^+^, CD68^+^, and CD3^+^ using EDTA retrieval. The standard Labeled Streptavidin Biotin approach was applied for detection with all antibodies. Following pretreatment and blocking steps, primary antibodies were incubated for 1 hour at room temperature. Biotinylated secondary antibodies were incubated for 30 minutes at room temperature (anti‐mouse Jackson Immuno 715‐066‐151 and anti‐rabbit Jackson Immuno 715‐066‐152) followed by a 15‐minute incubation with streptavidin‐HRP (DAKO P039701‐2). Visualization was achieved with DAB+application (DAKO DAB+K346811‐2). All slides were counterstained with Modified Mayer's Hematoxylin (DAKO S3309330‐2) and blued with 0.1% ammonium water and mounted with synthetic mounting media. Omission of primary antibody served as a negative control. Antibodies were tested and validated for IHC by the Histology Core Facility at Children's Hospital of Wisconsin (PEN, Director) ^23^. The IHC images were digitally recorded using a high‐resolution, whole slide scanner (NanoZoomer HT 2.0, Hamamatsu, Japan) at 40× magnification, and the data were reviewed using NDPview (version 2.7.43, Hamamatsu, Japan) for virtual image exploration. The scanned images were imported in the Image Analysis Software, (Visiopharm, Denmark) and analyzed at 20× magnification to quantify DAB expressions. The software was trained to capture DAB‐positive areas and the kidney tissue area by a preset thresholding and the linear Bayesian classification. The processed image is pseudo color coded for DAB‐positive area and tissue area for each kidney section are presented and the data are expressed as a percentage value. The IHC images were recorded and analyzed at the Children's Research Institute Imaging Core at the Medical College of Wisconsin.

#### 2.5.6 Flow cytometry assay

Circulating mononuclear cells and kidney tissues were obtained as described previously.^24^ Flow cytometry for CD3^+^ cells and CD45^+^ cells was performed as described previously.^24^

#### 2.5.7 Echocardiography

Left ventricular dimensions at end diastole were measured using echocardiography after irradiation of two kidneys and compared with sham‐irradiated rats. Prior to performing the ultrasound techniques, rats were anesthetized with isoflurane (3% for induction, 1‐2% for maintenance). The rat was placed in dorsal or lateral recumbency on a heated blanket to maintain normothermia, and the hair on the chest, legs, and arms removed using a depilatory agent (Nair) so that the EKG leads and the transducer contacted the skin directly. After hair removal, ultrasound transmission gel was applied to the chest for the echocardiogram. The operator was a professional sonographer experienced in rat echocardiography who was double blinded with respect to treatment allocation. An echocardiograph Vivid 7 (General Electric, Waukesha, Wisconsin, USA) was used with a M12L (11‐MHz) linear‐array transducer. Closed‐chest imaging was performed in the short‐axis view at the mid‐LV level (level of papillary muscles). The image depth was 2.5 cm and acquisition was at 236 frames/second with electrocardiographic gating. Left ventricular, right ventricular, and septal dimensions were measured after irradiation of the kidneys and in sham‐irradiated rats. The images were processed using EchoPAC Q analysis software (General Electric). The method has been previously described.^5^

#### 2.5.8 Statistical analysis

All values were expressed as the mean ± standard deviation, unless otherwise noted. Statistical analyses were performed using SigmaPlot^®^ version 11.0 software. Exploratory analyses were performed using the Shapiro–Wilk test^25^ followed by ANOVA with or without post hoc all pairwise multiple comparison procedures (Holm–Sidak method). The unpaired Student's *t* test was used for two‐group comparisons. The individual tests used are reported in the figure legends. Statistical analysis for the metabolite studies is described in “Metabolomic studies.” The threshold for statistical significance (*P* < .05) was prospectively identified and not varied in the data analysis.

## 3 RESULTS

### 3.1 Irradiation of one kidney with 10 Gy of x‐rays is sufficient to injure the kidney and increase systemic blood pressure

**FIGURE 1:**
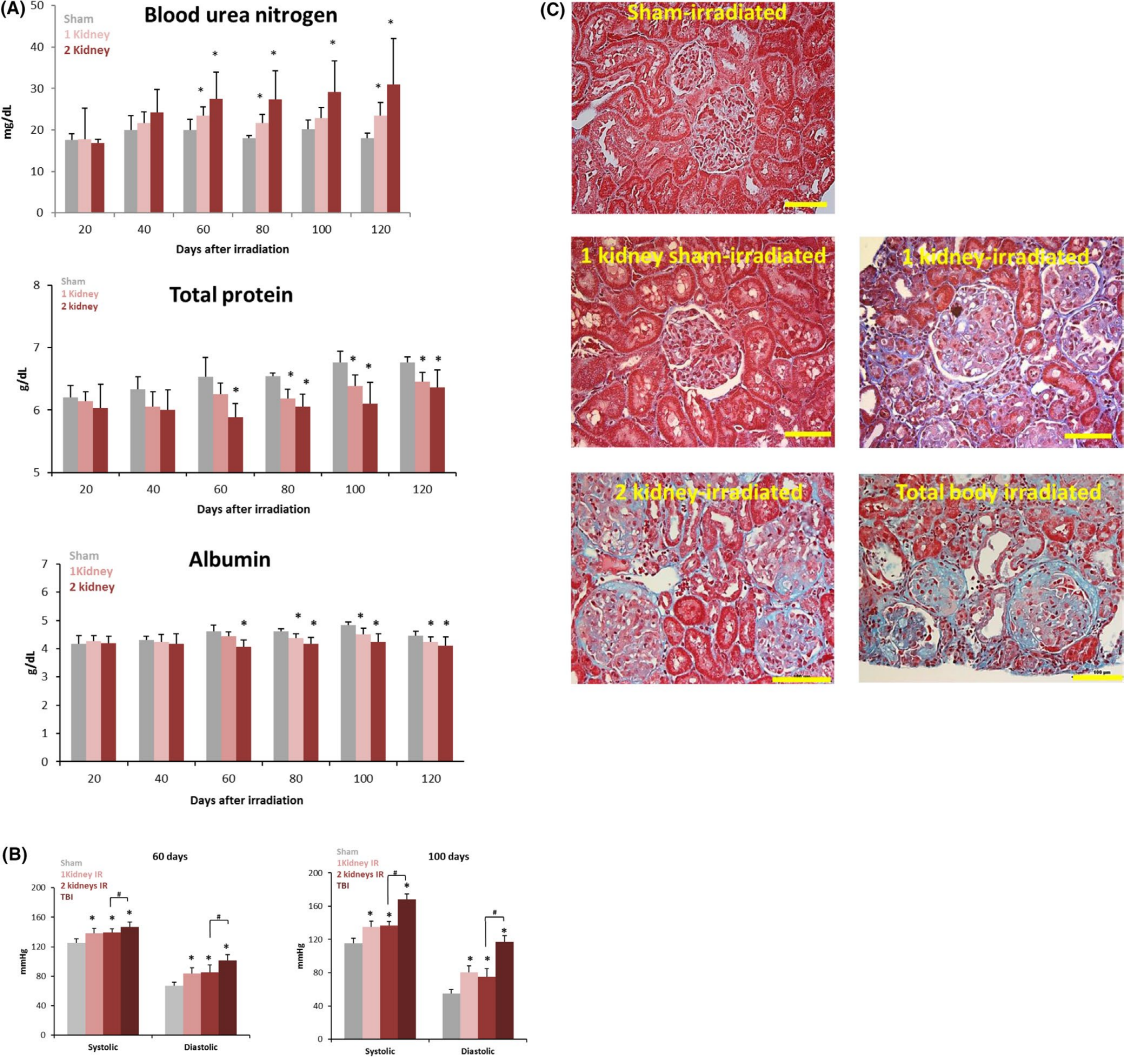
A, Blood urea nitrogen, total protein, and albumin levels in circulation after 10 Gy local kidney irradiation of WAG rats. Data are mean +*SD*. n = 5‐6 rats per group. *P* =< .05 vs sham‐irradiated control. B, Systemic blood pressure in WAG rats 60 and 120 after irradiation of one kidney, two kidneys, and the whole body. Data are mean +*SD*, n = 5‐6 animals per group. * = *P* < .05 vs sham‐irradiated control. # = *P* < .05, two‐kidney irradiation vs. whole body irradiation. C, Morphologic changes for kidney 120 days after irradiation of one kidney, two kidneys, and the whole body. Sections of kidney stained with trichrome. The horizontal scale bar represents a distance of 100 microns. Images are representative data from eight animals per group. Statistical analysis performed using the *t*‐test.

Prior studies showed that TBI with 10 Gy of X‐rays resulted in radiation nephropathy.^5^ We tested whether local irradiation of a single kidney or both the kidneys with a dose of 10 Gy would result in persistent radiation nephropathy. These initial studies used the WAG strain of rats using an anterior–posterior field. BUN levels remained stable over the 120‐day duration of the study in sham‐irradiated rats. Elevated BUN levels are a clear indication of kidney injury. BUN increased at 60 days after local irradiation of one kidney from a collimated X‐ray source and remained elevated at 120 days (Figure 1A). The increase in BUN was greater after irradiation of both the kidneys compared with irradiation of a single kidney, but the time needed for BUN levels to change was the same regardless of whether one or two kidneys were exposed. Total protein levels in the serum increased slightly but not significantly, and albumin levels remained stable over the duration of the study in sham‐irradiated rats. There were no changes in BUN, total protein or albumin in irradiated rats prior to 60 days. Notably, total protein and albumin each decreased at 60 days after local irradiation of one kidney or both the kidneys with a dose of 10 Gy and remained decreased 120 days after exposure (Figure 1A).

Diastolic and systolic blood pressure remained stable over the duration of the study in sham‐irradiated rats. Elevated blood pressures can indicate kidney injury as well as more widespread damage to blood vessels. Diastolic and systolic blood pressures were increased at 60 days after local irradiation of one kidney (Figure 1B). Blood pressures were increased further after irradiation of both the kidneys or the total body. The increase in blood pressure after irradiation of one kidney, both kidneys, or the whole body persisted 100 days after irradiation (Figure 1B).

**FIGURE 2:**
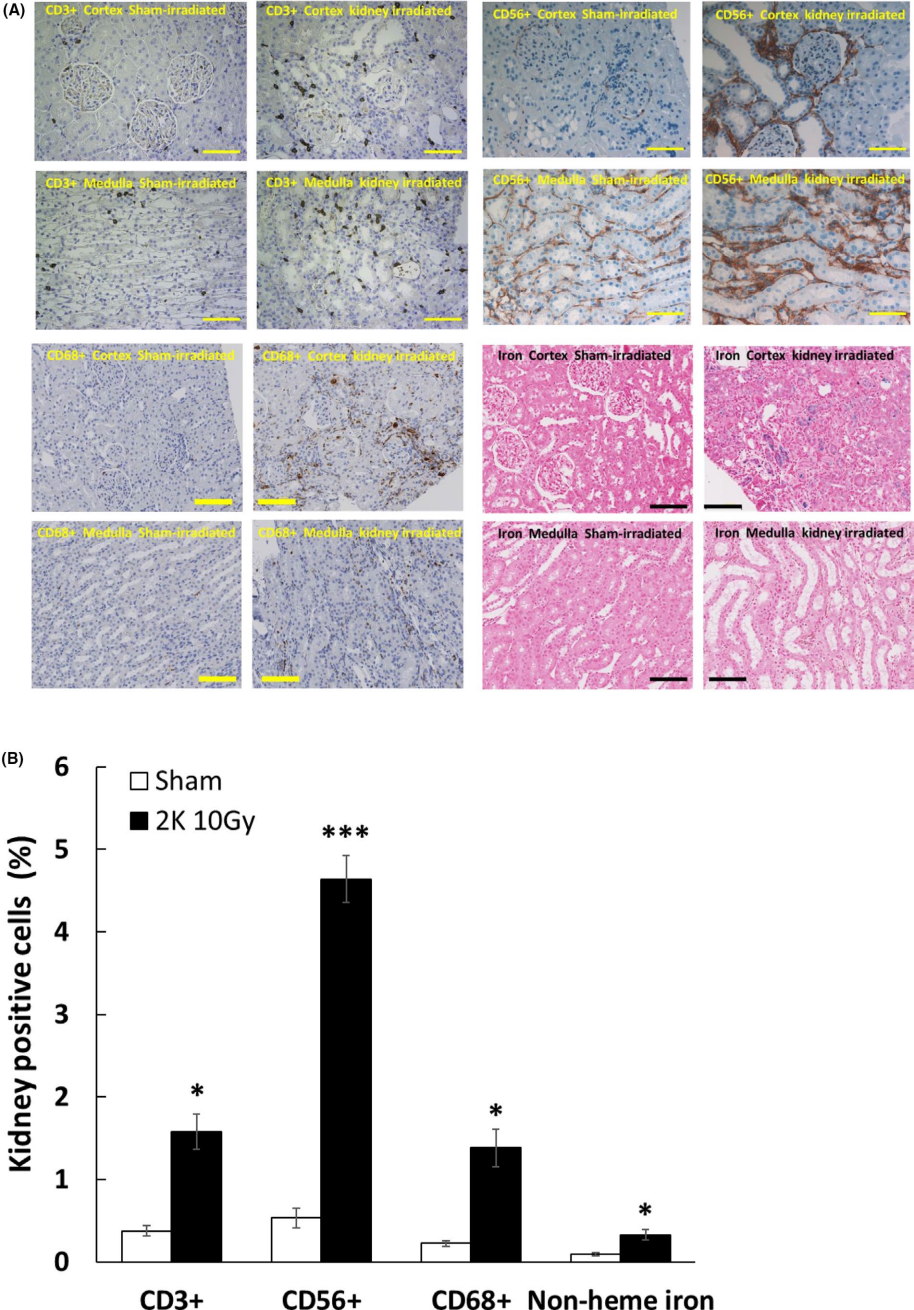
Injury to the kidney in WAG rats at 120 days after local kidney irradiation with 10 Gy X‐rays. A, T cells (CD3+), natural killer cells (CD56+), and macrophages (CD68+) appear as brown color. Iron infiltration kidneys after local irradiation was identified by hemosiderin pigment present in tubular epithelial cells, indicative of vascular coagulopathy. The horizontal scale bar represents a distance of 100 microns. Images are representative data from 3‐4 animals per group. B, Quantification of immune cells in cortex and medulla of kidney. Data are mean +SEM, n = 3‐4 animals per group. *=*P* < .05 vs sham‐irradiated control. ***=*P* < 0.01 vs sham‐irradiated control. Statistical analysis was performed by one‐way ANOVA and post hoc Holm–Sidak test

Histologic sections were analyzed by two pathologists (PEN and RK) blinded to specimen identity. Renal injury (glomerulosclerosis and fibrosis) was present 120 days after local irradiation of one kidney, both the kidneys, or the total body (Figure 1C). Hyaline material was deposited and sclerosis present in the capillary walls. There was no histologic evidence of injury in the nonirradiated kidney where the contralateral kidney was irradiated or in sham‐irradiated controls. Hemosiderin (an iron‐storage complex) deposition was increased after local kidney irradiation in tubular epithelial cells, indicative of microvascular injury and coagulopathy (Figure 2A).

### 3.2 Immune cells infiltrate the kidney after irradiation

To demonstrate the role of the immune system in the response of the kidney to local irradiation, IHC was performed on kidney tissue from WAG rats either sham‐irradiated or irradiated with 10 Gy using a lateral field. Local kidney irradiation resulted in increased infiltration of T lymphocytes (CD3^+^), natural killer cells (CD56^+^), and macrophages (CD68^+^) and into the cortex and medulla of the kidney after 120 days (Figure 2A), a time when radiation nephropathy was present. The extent of infiltration following irradiation was greatest for natural killer cells (Figure 2B).

### 3.3 Local irradiation of both the kidneys remodels the nontargeted heart

**FIGURE 3:**
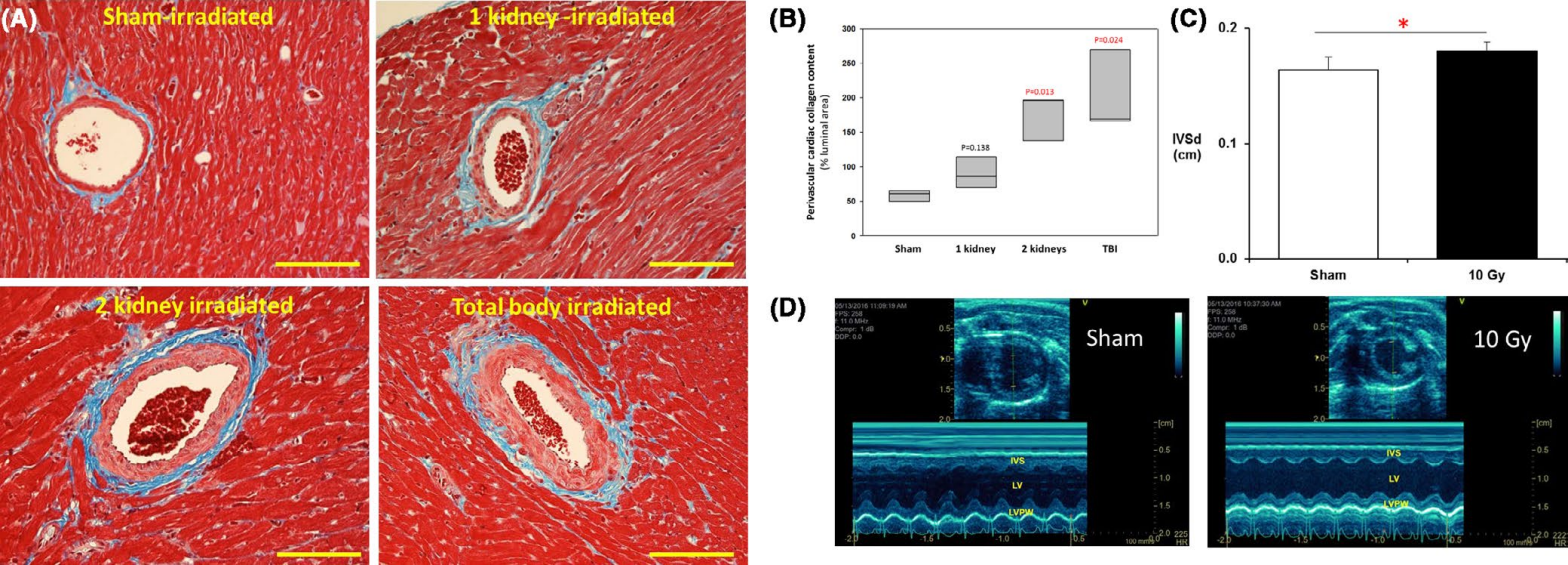
Cardiac remodeling in WAG rats after irradiation. A, Representative images of perivascular collagen deposition 120 d after irradiation of one kidney, two kidneys, and the total body. Sections of heart stained with trichrome. The horizontal scale bar represents a distance of 100 microns. B, Quantification of cardiac fibrosis present after irradiation of one kidney, both the kidneys, and the total body. Data are mean, median (25%), and median (75%), n = 3 animals per group. *P* values are shown vs sham‐irradiated control. C, Ventricular dimensions 120 d after irradiation of two kidneys. Interventricular wall thickness at end diastole increased after irradiation of two kidneys compared with sham‐irradiated rats, indicative of early onset left ventricular hypertrophy. Data are mean +*SD*, n = 5‐6/group. *=*P* < .05 vs sham. D, Representative echocardiogram images of sham‐irradiated and two kidney‐irradiated rats. Statistical analysis was performed by one‐way ANOVA (panel B) or *t* test (panel C)

To determine whether local irradiation of the kidneys results in remodeling of the nontargeted heart, one kidney, or two kidneys in male WAG rats were irradiated with 10 Gy of X‐rays using a lateral field. There were no deaths in WAG rats. Cardiac perivascular collagen deposition, a hallmark of radiation injury to the heart^5^ was increased 120 days after either local irradiation of both the kidneys or after the whole body compared with sham‐irradiated rats (Figure 3A). There was a small increase in cardiac perivascular collagen deposition after irradiation of one kidney that did not reach statistical significance. Trichrome staining revealed peri‐arterial fibrosis and irregular collagen deposition around the coronary vessels. Quantitative morphometric analysis to measure the extent of perivascular collagen deposition confirmed the histopathologic findings of a progressive increase in fibrosis in the groups receiving irradiation to one kidney, both kidneys, and the total body (Figure 3B). Gross examination of myocytes in left ventricle and interventricular septum in hearts from both kidney and total body‐irradiated rats showed a slightly enlarged appearance. To determine whether irradiation of the kidneys in WAG rats alters ventricular dimensions, we performed echocardiographic measurements. Local kidney irradiation of two kidneys increased inter ventricular septum thickness at end diastole compared with sham‐irradiated controls (Figure 3C,D).

### 3.4 Genetic deletion of *CD247* mitigates radiation nephropathy

**FIGURE 4:**
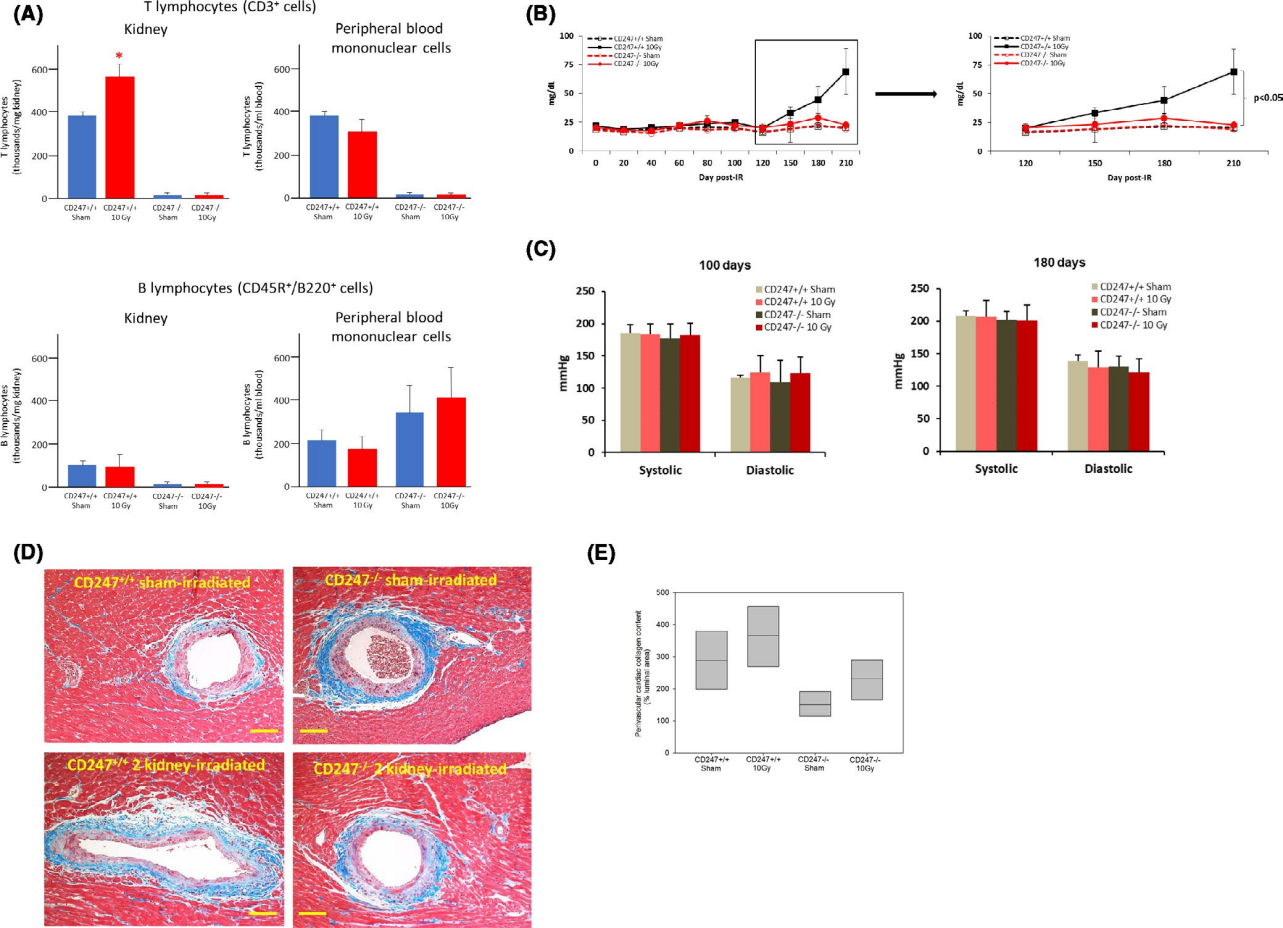
Genetic deletion of *CD247* in Dahl SS rats mitigates radiation nephropathy. A, Numbers of T lymphocytes (CD3+) and B lymphocytes (CD45R+/B220+) infiltrating the kidneys and present in the peripheral blood in sham‐irradiated and irradiated CD247+/+ and CD247−/− rats at 230 d after sham irradiation and irradiation of both the kidneys with 10 Gy, n = 3‐5/group (Panel A) B. Blood urea nitrogen levels in the peripheral circulation in irradiated and in sham‐irradiated CD247+/+ and CD247−/− rats over the full experimental period from 0‐210 d, and over the period from 120 to 210 d when BUN levels are increased in irradiated CD247+/+ rats (Panel B). There was no increase in BUN levels after irradiation of both the kidneys in CD247−/− rats, n = 6‐8/group. * = *P* < .05 vs sham. C, Systemic blood pressure in Dahl SS rats 100 and 180 d after sham irradiation and irradiation the two kidneys, n = 5‐8/group. There were no differences between groups (Panel C). D, Perivascular collaged deposition in sham‐irradiated and irradiated CD247+/+ and CD247−/− rats. Sections of heart stained with trichrome. The horizontal scale bar represents a distance of 100 microns (Panel D). E, Quantification perivascular collaged deposition. Data are mean, median (25%), and median (75%), n = 3 animals per group. There were no differences between groups (Panel E). Statistical analysis was performed by one‐way ANOVA (Panel B) or *t* test (Panel A, Panel C)

Results from the immunochemistry studies indicated a possible role for T cells in the response of the kidney to irradiation. To study the functional role of T cells in mediating radiation nephropathy, we used a genetic model of immune cell deficiency that lacks functional T cells. The *CD247* gene encodes the CD3 ζ chain involved in assembly, expression, and signal transduction of the T cell receptor complex.^26^ Deletion of *CD247* from the Dahl SS genetic background results in the absence of CD247 in the thymus and >99% reduction in circulating CD3+ T lymphocytes as compared with CD247^+/+^ littermates, and attenuates salt‐sensitive hypertension.^27^ To examine whether T cells infiltrating the kidney play a role in radiation nephropathy, the kidneys of wild‐type Dahl SS *CD247* ^+/+^ and Dahl SS *CD247* ^−/−^ knockout rats were irradiated with 10 Gy of X‐rays using a lateral field. Sham‐irradiated and irradiated rats from both the Dahl SS *CD247* ^+/+^ and Dahl SS *CD247* ^−/−^ exhibited mortality that was not statistically significant (data not shown). In sham‐irradiated Dahl SS *CD247* ^−/−^ rats, T cells (CD3+) were essentially depleted in the kidneys and peripheral blood mononuclear cells (Figure 4A, top). The levels of B cells (CD45R^+^/B220^+^) in the kidney and in peripheral blood mononuclear cells from Dahl SS *CD247* ^−/−^ rats were no different than the levels in Dahl SS *CD247* ^+/+^ rats (Figure 4A, bottom). Local irradiation of both the kidneys in Dahl SS *CD247* ^+/+^ rats resulted in elevated BUN levels after 120 days (Figure 4B, left panel). Genetic deletion of T cells in the irradiated Dahl SS *CD247* gene knockout rat mitigated increased BUN levels as compared with sham‐irradiated controls (Figure 4B, right panel). However, systolic and diastolic blood pressures were 1.6 and 2.1 times higher in sham‐irradiated Dahl SS rats (Figure 4C) than in WAG rats (Figure 1B), respectively. Irradiation of both the kidneys in CD247^+/+^ and CD247^−/−^ rats did not increase systemic blood pressure further (Figure 4C). Moreover, in sham‐irradiated Dahl SS *CD247* ^+/+^ rats (Figure 4E), perivascular collagen content was 4.8 times the level present in sham‐irradiated WAG rats (Figure 3B). There were no differences in cardiac perivascular collagen deposition between sham‐irradiated and irradiated CD247^+/+^ and CD247^−/−^ rats (Figure 4D,E), in contrast to what we observed with the WAG rats where kidney irradiation did result in the adverse cardiac outcome of increased perivascular collagen deposition (Figure 3B).

### 3.5 Metabolomics following local kidney irradiation reveals a broad effect on systemic metabolites in the circulation

**FIGURE 5:**
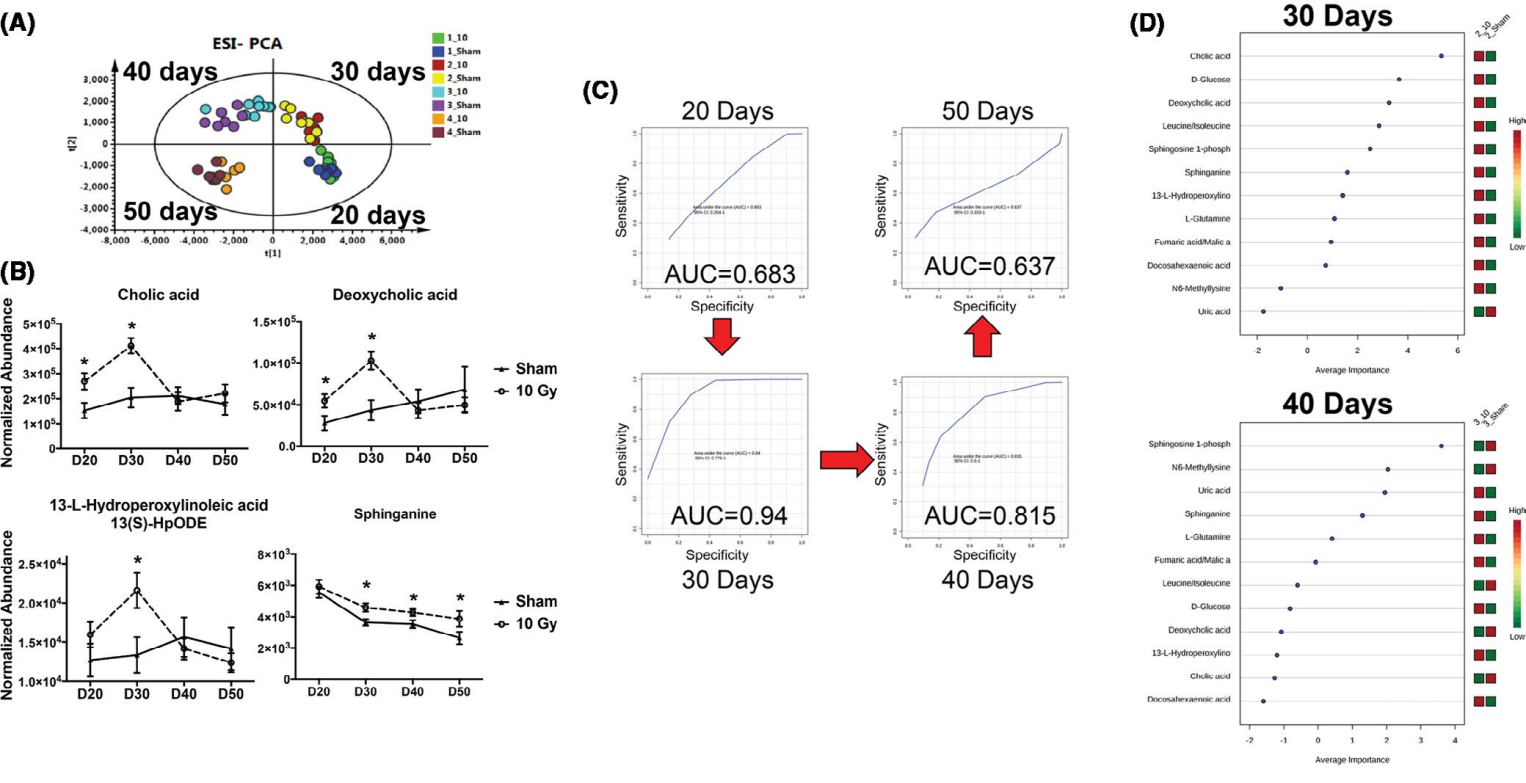
Metabolomic changes, predictive ability and biomarker importance in blood at 20, 30, 40, and 50 d post two‐kidney irradiation in WAG rats (n = 8/group). Panel A: Multivariate data analysis reveals a time‐dependent separation between sham and irradiated samples. Panel B: Examples of bile acids, pro‐inflammatory, and sphingolipid mediators. All four show significant changes at day 30 postirradiation. Data are presented as mean ±*SEM*, n = 8/group Asterisks represent a *P*‐value of <.05. Panel C: ROC curves based on a panel of 12 metabolites show high specificity and sensitivity of the signature at days 30 and 40 after irradiation. Panel D: Mean importance ranking of the 12 validated metabolites. Bile acids rank higher at 30 d, with a shift to energy and sphingolipid related metabolites rank at day 40. Eight rats were used for each experimental condition

Injury to the kidney of male WAG rats appears >60 days after local kidney irradiation with 10 Gy. Changes in blood components before 50 days can therefore reflect radiation damage to the kidney that can contribute to remodeling of the nontargeted heart. Untargeted metabolomics and multivariate data analysis showed a time‐dependent shift in metabolic profiles (Figure 5A) between sham and local kidney‐irradiated groups, indicative of divergent circulating molecules. Volcano plots at days 20, 30 40, and 50 after irradiation of both the kidneys showed a progressive increase of statistically significant ion perturbations, with the effect dissipating by day 50. This is consistent with the PCA scores plot (Figure 5A), in which separation between irradiated and nonirradiated samples at day 50 was also minimal (Figure S1).

Twelve biomarkers were chosen to be validated through tandem mass spectrometry (Table S1). Fumaric acid and malic acid shared common fragments, in addition to identical *m*/*z* and retention time, and their positive identification was not feasible, therefore they were reported together. Similarly, leucine and isoleucine were reported together. Four biomarkers (Figure 5B) highlight the potential early involvement of bile acids in radiation‐mediated cardiovascular function (cholic acid and deoxycholic acid) and of lipid mediators [13(S)‐HpODE and sphinganine] contributing to radiation related pro‐inflammatory signaling, and serve as indicators of increased apoptosis and damage to plasma membranes. Additional circulating metabolites that were identified include uric acid, N(6)‐methyllysine, glutamine, docosahexaenoic acid, and α‐D‐glucose. Multivariate ROC curves showed that the panel of 12 metabolites was able to distinguish between irradiated and nonirradiated groups with a high degree of sensitivity and specificity at 30 and 40 days postirradiation (AUC’s of 0.94 and 0.815; respectively; Figure 5C). Ranking of the metabolites by mean importance at 30 and 40 days after irradiation indicated a shift in top importance between bile acid‐related metabolites to sphingolipid metabolism (sphingosine‐1‐phosphate and sphinganine) and energy‐related intermediates (L‐glutamine, fumaric acid/ malic acid, leucine/isoleucine, α‐D‐glucose).

### 3.6 Local kidney irradiation results in a cascade of cytokine release into the circulation

**FIGURE 6:**
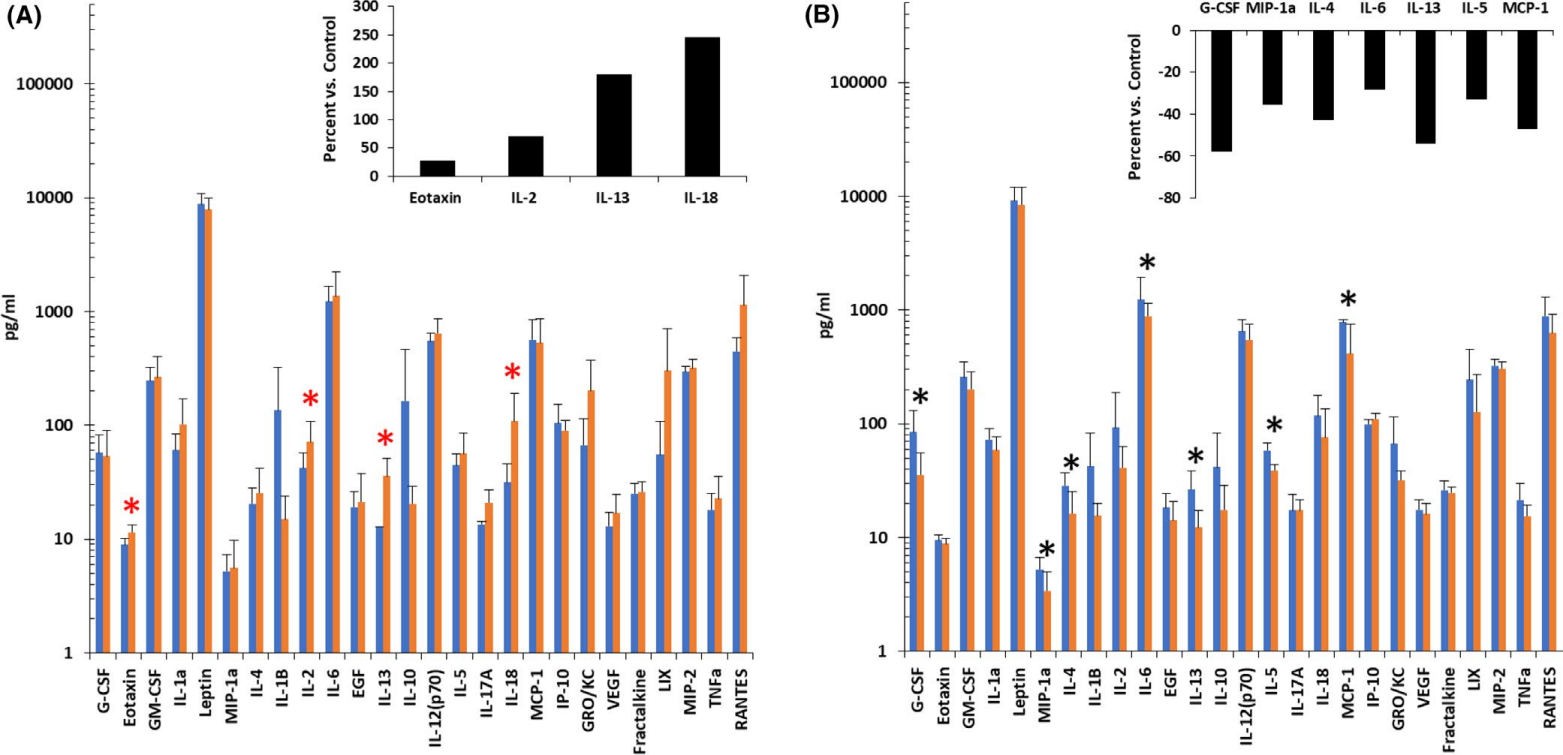
Cytokine changes in circulation post two‐kidney irradiation in WAG rats. Pro‐inflammatory cytokines increased at 20 d (Panel A) and decreased at 50 d (Panel B). Blue histograms show data from sham‐irradiated rats. Orange histograms show data from two kidney‐irradiated rats. Percentage changes are shown in black histograms. Data are mean +*SD*, n = 4/group. * =*P* < .05. Statistical analysis was performed using *t* test.

The levels of 27 cytokines in the serum from male WAG rats 20, 30, 40, and 50 days after local irradiation of both the kidneys were measured using a multiplex cytokine array to identify signaling molecules associated with the mechanism underlying remodeling of the nontargeted heart. Of the 27 cytokines measured, 26 could be reliably quantified. At 20 days after local kidney irradiation, the levels of four cytokines out of 26 detected were increased: Eotaxin (22%), IL‐2 (71%), IL‐13 (177%), and IL‐18 (241%) (Figure 6A). There were no decreases in any cytokines 20 days after local kidney irradiation. At 30 and 40 days after local kidney irradiation, there were no changes in the circulating levels of the 26 cytokines/chemokines. At 50 days after local kidney irradiation, the levels of seven out of 26 cytokines/chemokines detected were decreased: IL‐13 (56%), G‐CSF (58%), MIP‐1α (40%), IL‐4 (45%), IL‐6 (28%), IL‐5 (33%), and MCP‐1 (47%) (Figure 6B). Changes in IL‐13 levels occurred at both 20 and 50 days.

## 4 DISCUSSION

Results from the present study demonstrate local irradiation of the kidneys results in WAG rats results in a late pathology in the nontargeted heart, manifest as increased perivascular collagen deposition in the smaller diameter coronary vessels and increased interventricular septal thickness at end diastole, indicative of early onset left ventricular hypertrophy. The pathological changes in the heart are observed at a time when radiation nephropathy is concurrent. Injury to the kidney manifests as increased BUN, decreased total protein and albumin in the circulation, systemic hypertension, glomerulosclerosis, fibrosis, and hemosiderin deposition in the renal tissue. T lymphocytes, natural killer cells, and macrophages infiltrate the kidneys after irradiation, suggesting a role for the immune system in mediating injury. Genetic depletion of T cells mitigated radiation nephropathy after local kidney irradiation, supporting a role for the immune system in mediating injury to the kidney, raising the untested possibility that T cells may be the effector cells ultimately responsible for the cardiac remodeling in a susceptible strain of animals. Further studies are needed to examine the role of the relatively large numbers of natural killer cells present in the kidney following irradiation, and in the mechanism underlying the subsequent cardiac pathology. Release of pro‐inflammatory cytokines and low‐molecular weight metabolites after irradiation of the kidneys are associated with and may contribute to remodeling of the nontargeted heart in a susceptible rat strain such as WAG, implicating their likely role in the transmission of a signal between the kidney and the heart. The high baseline blood pressures and the elevated levels of cardiac fibrosis in the Dahl SS wild‐type animals likely prohibited the observation of any radiogenic cardiac remodeling in this strain.

Metabolomic changes showed a delayed response after local kidney irradiation in WAG rats, with the highest peak of select biomarkers in the circulation present at days 30 and 40 postexposure. These biomarkers have a direct link to inflammatory processes ^28^, ^29^, ^30^ and endogenous processes such as apoptosis (13(S)‐HpODE, docosahexaenoic acid, sphinganine, sphingosine‐1‐phosphate), while others indicate a dysfunctional tricarboxylic acid or active urea cycle, suggesting that dysregulation of mitochondria is involved in the development of kidney injury after irradiation (fumaric acid/malic acid, glutamine, and glucose).^31^ The decrease in circulating bioavailability of N(6)‐methyllysine, a normal metabolite found in biofluids, may be an indicator of increased epigenetic regulation that can be further explored in the future. One of the most interesting findings, however, was the initial increase of the bile acids cholic acid and deoxycholic acid. Although the role of the kidney in modulating changes in the levels of circulating bile acids is minimal compared to contributions from the gastrointestinal tissue or the liver, kidney dysfunction has been shown to lead to increased circulating bile acids.^32^ A connection between bile acids and cardiovascular disease has also been uncovered, with increased serum bile acids leading to cardiomyopathy and cardiac dysfunction.^33^ Bile acids can alter vascular dynamics,^34^ and pharmacological intervention to block bile acid receptors has been suggested as a method to decrease dyslipidemia and therefore cardiovascular disease.^35^ Further research into these areas to identify connections between these metabolites and the development of the phenotype in WAG *CD 247* knockout rats is warranted to discern whether these metabolic alterations may be mediated by T cells.

We measured cytokines at 20, 30, 40, and 50 days following kidney irradiation to detect whether patterns of response of a pro‐inflammatory nature can recur in a cyclical fashion, as previous studies have shown this over succeeding days, weeks, and months after irradiation,^36^ forming a persistent cascade of cytokines.^37^ Local kidney irradiation increases the levels of several cytokines in the circulation at 20 days postexposure, followed by a quiescent period at 30 and 40 days when there are no changes in circulating cytokine levels or increases in systemic blood pressure, followed by a decrease in circulating cytokines at 50 days. These time points were chosen to identify changes in cytokines that occur before evidence of injury to kidney tissue. Elevation of the levels of eotaxin, IL‐2, IL‐13, and IL‐18 at 20 days following kidney irradiation precede that of BUN. These cytokines may be potential biomarkers for the onset of radiation nephropathy that drive remodeling in the nontargeted heart, and therapeutic targets. Twenty days after local kidney irradiation, the inflammatory cytokine IL‐18 was elevated the most of all cytokines measured in the circulation. Elevated IL‐18 levels in the circulation predict cardiovascular mortality in patients with kidney disease.^38^ Further studies are needed to identify the pathways activated by kidney irradiation whereby metabolites and cytokines drive remodeling of the nontargeted heart.

Kidney injury did not occur for at least 60 days following radiation. Some of the biological processes present before the appearance of radiation injury to kidney in WAG rats are known. Expression of p21, a gene that senses DNA damage, was upregulated 13‐24 fold at 1, 7, 21, and 49 days following 10 Gy total body irradiation (unpublished observation). Genes more specifically associated with initiating and maintaining kidney injury following other stressors were not expressed in kidney 1‐49 days following 10 Gy total body irradiation.^39^ It is possible that gene expression associated with kidney injury occurs later than 49 days and before 60 days when kidney injury is present in the current study, but this is considered unlikely. Identifying relevant changes present in the kidney prior to the appearance of injury would be a key to understanding the biological process. However, the underlying mechanism leading to injury in the kidney remains unknown. Future studies are needed to understand the delay in kidney injury following irradiation. The RAAS system plays a crucial role in blood pressure regulation. Suppression of the RAAS system using ACE inhibitors and AT1 blockers mitigates radiation‐induced renal injury. However, hypertension of radiation nephropathy is not aldosterone dependent,^40^ with no clear evidence to date of activation of the RAAS system upon irradiation of kidney in WAG/RijCmcr rats.^41^

The response to a 10 Gy dose of radiation absorbed by the kidneys showed a delayed increase in BUN in Dahl SS rats compared with WAG rats. Strain differences are present in the response of rodents to radiation. C57BL/6 mice are more susceptible to pulmonary fibrosis compared with C3H/HeJ and CBA/J mice following irradiation of the thorax.^42^ Sprague Dawley and Fischer 344 rats exhibit pulmonary dysfunction following hemi thoracic radiation. Pulmonary function in the Wistar strain was unaffected by hemi thoracic radiation.^43^ Spontaneously hypertensive rats but not Wistar Kyoto rats exhibit increased systolic blood pressure following TBI.^44^ Taken together, these strain‐dependent differences in the response to radiation suggests there is an underlying genetic basis for the observed change in the BUN phenotype.

Systolic and diastolic blood pressures were 1.6 and 2.1 times higher in sham‐irradiated Dahl SS rats (Figure 4C) than in WAG rats (Figure 1B), respectively. Perivascular cardiac collagen content in sham‐irradiated wild‐type Dahl SS rats (Figure 4E) was 4.8 times higher than in WAG rats (Figure 3B). Irradiation of both the kidneys increased systemic blood pressure and cardiac perivascular collagen content in WAG rats (Figures 1B and 3B) but did not increase systemic blood pressure or perivascular collagen content further in Dahl SS rats (Figure 4C,E). These findings show systemic hypertension and cardiac fibrosis, hallmarks of radiation injury, are already present in sham‐irradiated Dahl SS rats at levels that likely obscure any additional effect of irradiation. The genetic background of the Dahl SS rat may harbor genes that constitutively express disease phenotypes including cardiac fibrosis and systemic hypertension. Thus, Dahl SS rats may not be a suitable model for investigations of radiogenic hypertension and cardiac fibrosis.

Radiation therapy to treat cancers in pelvis or abdomen inevitably involves the exposure of normal tissues in the path of the beam of photons^45^ and the tissue surrounding the tumor. Radiotherapy for pelvic or abdominal cancers can injure the normal kidneys. Even with kidney‐sparing methods during radiotherapy for cervical cancer, the absorbed dose to the kidney can reach 10 Gy,^7^ a dose that can cause renal injury.^8^, ^9^ Information from studies on damage to normal tissues from radiation may be applied to other cancer therapies and to accidental or intentional radiation exposure.^46^

In experimental studies done by others, irradiation of the lower abdomen with 10 Gy of gamma rays in mice was shown to upregulate nuclear factor kappa B signaling in the nontargeted heart as early as 1 day after irradiation.^47^ This study did not evaluate time points after 1 day, or physiological changes in the lower abdomen and heart. Irradiation of one organ resulting in injury to another organ is not exclusive to heart. Collimated irradiation of the rat liver with X‐rays has been associated with distal effects in the brain manifested as behavioral changes.^48^ Irradiation of the rat head with densely ionizing 1100 MeV titanium ions elicited altered protein expression in the nontargeted liver many months postirradiation.^49^ Irradiation of the brain with 20 Gy of gamma rays resulted in degenerative changes in the coronary arteries after 180‐720 months.^50^ In another study with densely ionizing ^56^Fe ions, irradiation of mouse orbital regions with 0.1 or 0.2 Gy of ^56^Fe ions resulted in degenerative changes in coronary arteries.^51^ Taken together, these studies suggest that both sparsely ionizing radiation (X‐ or gamma rays) and densely ionizing radiation (e.g., Ti or Fe ions) of individual organs exerts an effect on multiple nontargeted organs, including the heart. Nontargeted effects are not restricted to ionizing radiation. Kidney ischemia/reperfusion injury results in infiltration of T cells in the lung.^52^

Kidney disease is a known risk factor for the development of cardiac disease.^53^, ^54^ Our findings suggest that radiotherapy to lower hemi body organs can cause modest but significant elevations in cardiac perivascular collagen content in the sensitive WAG rat strain.^5^ In support of this notion, long‐term survivors of testicular cancer who received infradiaphragmatic radiotherapy are at increased risk for cardiac disease (RR = 2.40, 95% CI 1.04 to 5.45, *P* = .036).^55^ Mean dose received by the heart in this cohort was estimated to have been 0.75 Gy, with only 14% of the cardiac volume receiving over 0.9 Gy. This dose of cardiac radiation is unlikely to account for the observed elevation in risk. Irradiation of renal tissue in the planned treatment volume may have led to hypertension and a consequent increase in the risk of cardiac disease in these patients. Others showed that infradiaphragmatic radiotherapy for testicular cancer increased serum markers of inflammation and endothelial cell dysfunction.^56^ We hypothesize that radiotherapy initiates self‐perpetuating endothelial inflammation that confers adverse risk to the whole cardiovascular system rather than just the parts directly irradiated. If true, this could explain how large volume infradiaphragmatic radiotherapy increases risk without significant cardiac irradiation. Thus, persons receiving large volume abdominal irradiation for other cancers may also increase cardiac risk.

The findings of the present basic science study demonstrate radiation‐induced cardiac remodeling can be indirect, with lower hemi body organs such as the kidney exporting factors into the circulation that cause disease outside of the irradiated field in the shielded, nontargeted heart. This nontargeted effect appears to be mediated in part by the immune system. Our findings may reflect a facet of the systemic antitumor effects outside of the irradiated field mediated by the immune system initially described by Demaria and Formenti.^57^ Our findings help provide insight into why some cancer survivors who have been treated with radiation have an increased risk for serious heart disease later in life. The influence of radiotherapy on nontargeted organs will need to become an integral part of patient management posttreatment. Therapeutics targeting signals exported from the injured kidney into the circulation that initiate fibrosis in the nontargeted heart will need to be identified to help cancer survivors treated with radiation from developing serious and potentially life‐threatening cardiac disease.

## DISCLOSURES

None.

## CONFLICT OF INTEREST

The authors declare they have no competing interests.

## AUTHOR CONTRIBUTIONS

M. Lenarczyk and J. Baker designed research. M. Lenarczyk, E. Laiakis, A. Kronenberg, D. Mattson, D. Johnson, and J. Baker analyzed the data. M. Lenarczyk, P. North, R Komorowski, M Mäder performed research. M. Lenarczyk, A. Kronenberg, E. Laiakis, and J. Baker wrote the paper.

## Data Availability

Study data will be deposited in the NASA Life Sciences Data Archive (http://lsda.jsc.nasa.gov/) following publication.
