# Supplementary figures and images for: Irradiation of the kidneys causes pathologic remodeling in the nontargeted heart: A role for the immune system
Source: FASEB Bioadv. 2020 Oct 23;2(12):705–19. doi: 10.1096/fba.2020-00071 (PMC7734425; doi:10.1096/fba.2020-00071)

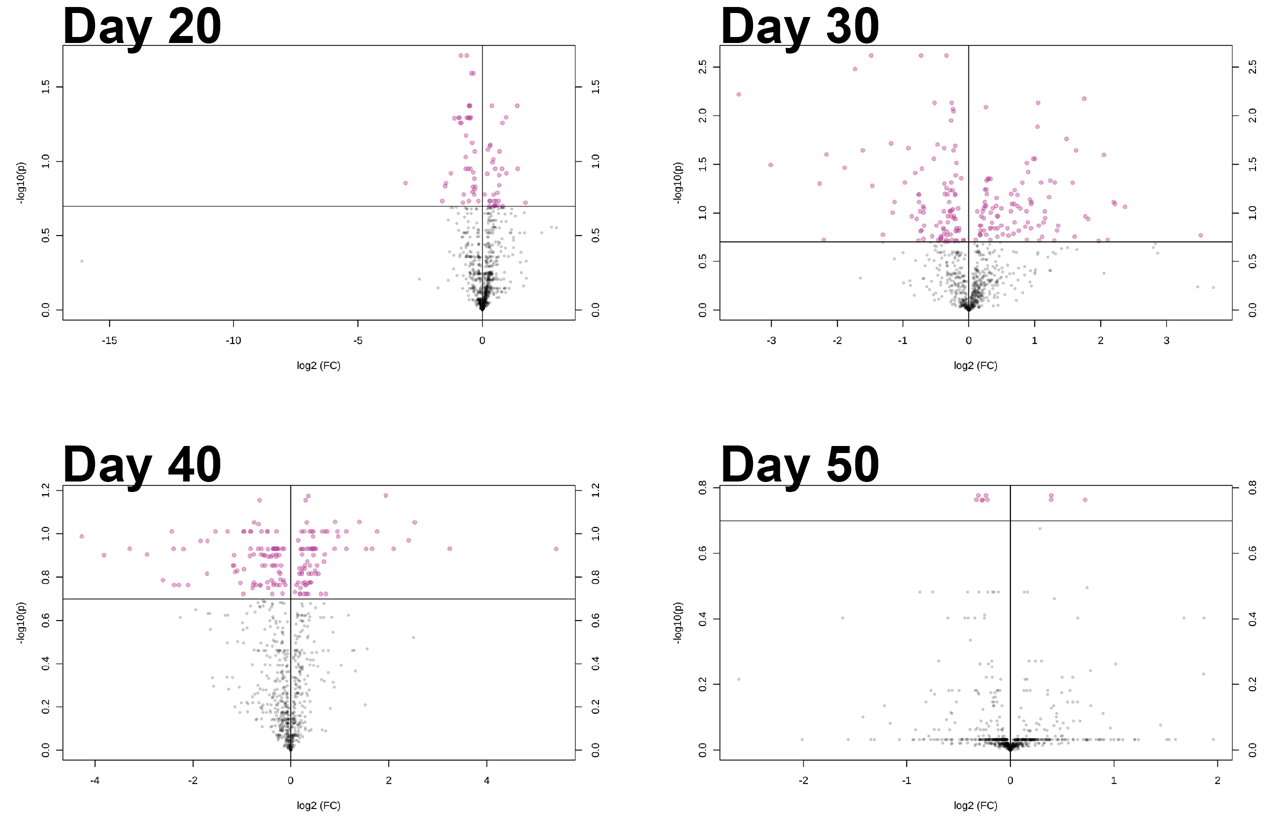

Supplement: Supplementary file 1 [file FBA2-2-705-s001.tif]
