# Supplementary material for: Irradiation of the kidneys causes pathologic remodeling in the nontargeted heart: A role for the immune system
Source: FASEB Bioadv. 2020 Oct 23;2(12):705–19. doi: 10.1096/fba.2020-00071 (PMC7734425; doi:10.1096/fba.2020-00071)
Supplement: Supplementary file 2 [file FBA2-2-705-s002.docx]

| **Supplementary Table 1: Metabolomic biomarkers** | |  |  |  |  |  |
| --- | --- | --- | --- | --- | --- | --- |
|  |  |  | **Day 20_Sham** | | **Day 20_IR** | |
|  | ***m/z*_Retention time** | **Adduct** | **Average** | **SEM** | **Average** | **SEM** |
| **Deoxycholic acid** | 391.2844_6.85 | **[M-H]-** | 32294.27 | 8650.27 | 54764.24 | 8004.69 |
| **D-Glucose** | 179.0556_0.31 | **[M-H]-** | 5505.07 | 218.56 | 4949.17 | 412.16 |
| **Cholic acid** | 407.2793_6.36 | **[M-H]-** | 175091.81 | 24653.82 | 269202.36 | 32752.63 |
| **Sphinganine** | 302.3058_6.89 | **[M+H]+** | 5596.14 | 403.27 | 5924.90 | 434.34 |
| **Sphingosine 1-phosphate** | 378.2405_6.93 | **[M-H]-** | 10146.09 | 559.24 | 9889.57 | 1022.37 |
| **Leucine/Isoleucine** | 130.0866_0.43 | **[M-H]-** | 6488.19 | 701.73 | 5440.29 | 1111.40 |
| **Fumaric acid/ Malic acid** | 115.0031_0.36 | **[M-H]-** | 3020.62 | 228.17 | 2822.23 | 253.98 |
| **Docosahexaenoic acid** | 327.2321_8.21 | **[M-H]-** | 28415.16 | 4695.36 | 26860.62 | 2308.84 |
| **13-L-Hydroperoxylinoleic acid [13(S)-HpODE]** | 311.2219_7.17 | **[M-H]-** | 14453.33 | 1321.48 | 15948.04 | 1638.06 |
| **L-Glutamine** | 145.0608_0.31 | **[M-H]-** | 29144.83 | 953.61 | 26739.15 | 904.80 |
| **N(6)-Methyllysine** | 159.1128_0.38 | **[M-H]-** | 25250.08 | 2058.55 | 24722.94 | 1964.04 |
| **Uric acid** | 167.0201_0.34 | **[M-H]-** | 5705.21 | 1925.02 | 11119.14 | 1803.97 |
|  |  |  |  |  |  |  |
|  |  |  | **Day 30_Sham** |  | **Day 30_IR** |  |
|  | ***m/z*_Retention time** | **Adduct** | **Average** | **SEM** | **Average** | **SEM** |
| **Deoxycholic acid** | 391.2844_6.85 | **[M-H]-** | 43579.79 | 12121.84 | 102636.85 | 10732.13 |
| **D-Glucose** | 179.0556_0.31 | **[M-H]-** | 2937.44 | 332.08 | 4899.17 | 506.13 |
| **Cholic acid** | 407.2793_6.36 | **[M-H]-** | 204852.68 | 39368.26 | 410866.49 | 30989.20 |
| **Sphinganine** | 302.3058_6.89 | **[M+H]+** | 3652.19 | 207.10 | 4594.54 | 266.42 |
| **Sphingosine 1-phosphate** | 378.2405_6.93 | **[M-H]-** | 21997.18 | 2139.62 | 27412.97 | 1535.20 |
| **Leucine/Isoleucine** | 130.0866_0.43 | **[M-H]-** | 2923.65 | 721.14 | 5629.17 | 512.63 |
| **Fumaric acid/ Malic acid** | 115.0031_0.36 | **[M-H]-** | 2910.60 | 262.37 | 3511.21 | 238.60 |
| **Docosahexaenoic acid** | 327.2321_8.21 | **[M-H]-** | 22688.28 | 2820.26 | 32093.11 | 3889.22 |
| **13-L-Hydroperoxylinoleic acid [13(S)-HpODE]** | 311.2219_7.17 | **[M-H]-** | 13349.00 | 2257.99 | 21615.49 | 2248.70 |
| **L-Glutamine** | 145.0608_0.31 | **[M-H]-** | 25693.54 | 1079.37 | 28845.31 | 635.23 |
| **N(6)-Methyllysine** | 159.1128_0.38 | **[M-H]-** | 25861.91 | 2081.65 | 25060.46 | 1154.30 |
| **Uric acid** | 167.0201_0.34 | **[M-H]-** | 9270.75 | 651.49 | 9285.04 | 654.77 |
|  |  |  |  |  |  |  |
|  |  |  | **Day 40_Sham** |  | **Day 40_IR** |  |
|  | ***m/z*_Retention time** | **Adduct** | **Average** | **SEM** | **Average** | **SEM** |
| **Deoxycholic acid** | 391.2844_6.85 | **[M-H]-** | 54239.12 | 14304.63 | 43456.57 | 9431.34 |
| **D-Glucose** | 179.0556_0.31 | **[M-H]-** | 4064.41 | 399.09 | 4330.55 | 363.86 |
| **Cholic acid** | 407.2793_6.36 | **[M-H]-** | 211980.89 | 34622.79 | 188919.69 | 36327.09 |
| **Sphinganine** | 302.3058_6.89 | **[M+H]+** | 3541.18 | 247.54 | 4284.51 | 220.39 |
| **Sphingosine 1-phosphate** | 378.2405_6.93 | **[M-H]-** | 21209.24 | 1127.84 | 16665.41 | 1476.88 |
| **Leucine/Isoleucine** | 130.0866_0.43 | **[M-H]-** | 3764.27 | 524.91 | 3542.15 | 292.75 |
| **Fumaric acid/ Malic acid** | 115.0031_0.36 | **[M-H]-** | 2356.02 | 413.28 | 3182.20 | 334.88 |
| **Docosahexaenoic acid** | 327.2321_8.21 | **[M-H]-** | 23885.88 | 4294.65 | 25621.27 | 2521.31 |
| **13-L-Hydroperoxylinoleic acid [13(S)-HpODE]** | 311.2219_7.17 | **[M-H]-** | 15645.97 | 2539.28 | 14162.01 | 1387.00 |
| **L-Glutamine** | 145.0608_0.31 | **[M-H]-** | 22163.88 | 471.15 | 23129.25 | 433.92 |
| **N(6)-Methyllysine** | 159.1128_0.38 | **[M-H]-** | 61170.87 | 2627.33 | 54902.78 | 3665.27 |
| **Uric acid** | 167.0201_0.34 | **[M-H]-** | 4128.28 | 510.85 | 5477.84 | 389.22 |
|  |  |  |  |  |  |  |
|  |  |  | **Day 50_Sham** | | **Day 50_IR** | |
|  | ***m/z*_Retention time** | **Adduct** | **Average** | **SEM** | **Average** | **SEM** |
| **Deoxycholic acid** | 391.2844_6.85 | **[M-H]-** | 61449.05 | 24458.37 | 49823.76 | 9185.79 |
| **D-Glucose** | 179.0556_0.31 | **[M-H]-** | 3395.18 | 352.76 | 3376.11 | 336.11 |
| **Cholic acid** | 407.2793_6.36 | **[M-H]-** | 178938.34 | 42526.06 | 221388.99 | 35281.80 |
| **Sphinganine** | 302.3058_6.89 | **[M+H]+** | 2643.56 | 400.94 | 3875.68 | 499.89 |
| **Sphingosine 1-phosphate** | 378.2405_6.93 | **[M-H]-** | 23208.73 | 1366.02 | 24440.88 | 1091.79 |
| **Leucine/Isoleucine** | 130.0866_0.43 | **[M-H]-** | 3123.36 | 253.39 | 2473.15 | 532.54 |
| **Fumaric acid/ Malic acid** | 115.0031_0.36 | **[M-H]-** | 828.79 | 222.50 | 1039.22 | 305.47 |
| **Docosahexaenoic acid** | 327.2321_8.21 | **[M-H]-** | 25838.27 | 3122.94 | 21513.84 | 2097.59 |
| **13-L-Hydroperoxylinoleic acid [13(S)-HpODE]** | 311.2219_7.17 | **[M-H]-** | 14171.58 | 2722.49 | 12382.20 | 1195.66 |
| **L-Glutamine** | 145.0608_0.31 | **[M-H]-** | 27171.39 | 1061.59 | 25870.61 | 812.56 |
| **N(6)-Methyllysine** | 159.1128_0.38 | **[M-H]-** | 39714.13 | 1810.22 | 29868.17 | 2731.72 |
| **Uric acid** | 167.0201_0.34 | **[M-H]-** | 3382.18 | 433.93 | 3292.46 | 782.02 |
