# Supplementary material for: Irradiation of the kidneys causes pathologic remodeling in the nontargeted heart: A role for the immune system
Source: FASEB Bioadv. 2020 Oct 23;2(12):705–19. doi: 10.1096/fba.2020-00071 (PMC7734425; doi:10.1096/fba.2020-00071)

Supplementary Table 1: Metabolomic biomarkers

			Day 20_Sham		Day 20_IR		Day 30_Sham		Day 30_IR		Day 40_Sham		Day 40_IR		Day 50_Sham		Day 50_IR	
	m/z _Retention time	Adduct	Average	SEM	Average	SEM	Average	SEM	Average	SEM	Average	SEM	Average	SEM	Average	SEM	Average	SEM
**Deoxycholic acid**	391.2844_6.85	[M-H]-	32294.27	8650.27	54764.24	8004.69	43579.79	12121.84	102636.85	10732.13	54239.12	14304.63	43456.57	9431.34	61449.05	24458.37	49823.76	9185.79
**D-Glucose**	179.0556_0.31	[M-H]-	5505.07	218.56	4949.17	412.16	2937.44	332.08	4899.17	506.13	4064.41	399.09	4330.55	363.86	3395.18	352.76	3376.11	336.11
**Cholic acid**	407.2793_6.36	[M-H]-	175091.81	24653.82	269202.36	32752.63	204852.68	39368.26	410866.49	30989.20	211980.89	34622.79	188919.69	36327.09	178938.34	42526.06	221388.99	35281.80
**Sphinganine**	302.3058_6.89	[M+H]+	5596.14	403.27	5924.90	434.34	3652.19	207.10	4594.54	266.42	3541.18	247.54	4284.51	220.39	2643.56	400.94	3875.68	499.89
**Sphingosine 1-phosphate**	378.2405_6.93	[M-H]-	10146.09	559.24	9889.57	1022.37	21997.18	2139.62	27412.97	1535.20	21209.24	1127.84	16665.41	1476.88	23208.73	1366.02	24440.88	1091.79
**Leucine/Isoleucine**	130.0866_0.43	[M-H]-	6488.19	701.73	5440.29	1111.40	2923.65	721.14	5629.17	512.63	3764.27	524.91	3542.15	292.75	3123.36	253.39	2473.15	532.54
**Fumaric acid/ Malic acid**	115.0031_0.36	[M-H]-	3020.62	228.17	2822.23	253.98	2910.60	262.37	3511.21	238.60	2356.02	413.28	3182.20	334.88	828.79	222.50	1039.22	305.47
**Docosahexaenoic acid**	327.2321_8.21	[M-H]-	28415.16	4695.36	26860.62	2308.84	22688.28	2820.26	32093.11	3889.22	23885.88	4294.65	25621.27	2521.31	25838.27	3122.94	21513.84	2097.59
**13-L-Hydroperoxylinoleic acid [13(S)-HpODE]**	311.2219_7.17	[M-H]-	14453.33	1321.48	15948.04	1638.06	13349.00	2257.99	21615.49	2248.70	15645.97	2539.28	14162.01	1387.00	14171.58	2722.49	12382.20	1195.66
**L-Glutamine**	145.0608_0.31	[M-H]-	29144.83	953.61	26739.15	904.80	25693.54	1079.37	28845.31	635.23	22163.88	471.15	23129.25	433.92	27171.39	1061.59	25870.61	812.56
**N(6)-Methyllysine**	159.1128_0.38	[M-H]-	25250.08	2058.55	24722.94	1964.04	25861.91	2081.65	25060.46	1154.30	61170.87	2627.33	54902.78	3665.27	39714.13	1810.22	29868.17	2731.72
**Uric acid**	167.0201_0.34	[M-H]-	5705.21	1925.02	11119.14	1803.97	9270.75	651.49	9285.04	654.77	4128.28	510.85	5477.84	389.22	3382.18	433.93	3292.46	782.02

Supplement Figure 1. Volcano plots at days 20, 30, 40 and 50. Volcano plots were constructed from combined ESI- and ESI+ data, graphed as –log10 FDR corrected p-value (FDR 0.2 cutoff) versus log2 fold change (no fold change cutoff).

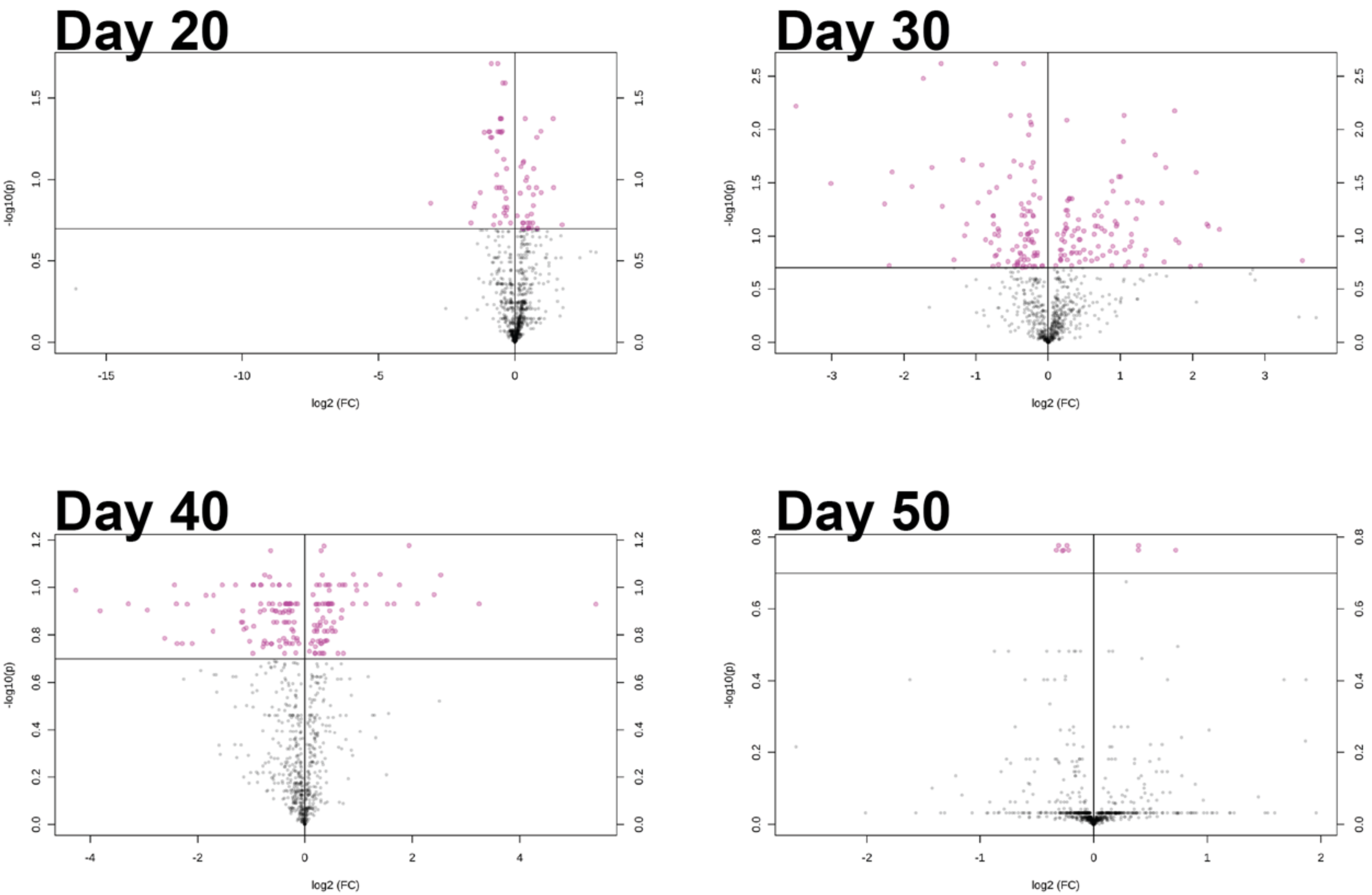

Supplement: Supplementary file 3 [file FBA2-2-705-s003.pdf]
